# Syringocystadenoma papilliferum in an unusual location*

**DOI:** 10.1590/abd1806-4841.20153862

**Published:** 2015

**Authors:** Bianca Angelina Macêdodo Nascimento, Clívia Maria Oliveira Carneiro, Alessandra Haber Carvalho, Maraya de Jesus Semblano Bittencourt, Marion Guimarães Drago, Lívia Karlla Marinho Freitas

**Affiliations:** 1 Universidade Federal do Pará (UFPA) - Belém (PA), Brazil

**Keywords:** Apocrine glands, Child, Infant, Neoplasms, adnexal and skin appendage, Vulva

## Abstract

Syringocystadenoma papilliferum is a rare benign hamartomatous adnexal tumor of the apocrine or eccrine sweat glands. Most patients present a solitary lesion in the head and neck region. Presentation outside the head and neck region is even more uncommon. We present a case of Syringocystadenoma papilliferum with papulonodular lesion located on the vulva of an infant girl. This case illustrates the atypical location of this rare disease and adds to the differential diagnosis of lesions on the vulva.

## INTRODUCTION

Syringocystadenoma papilliferum (SCAP) is a rare benign adnexial neoplasm, of controversial origin, derived from apocrine and eccrine glands.^1,2,3^ More common in children and adolescents, it is typically found on the scalp, neck and face, with few other locations reported. Histopathology is fundamental to exclude other dermatoses and define therapeutic approach.

## CASE REPORT

Female child, 8 years old, whose mother reported the onset of a nodule in the genital region since birth, with occasional pruritus, of slow and gradual growth. She underwent antibiotic therapy, corticosteroids and topical antifungals, with no improvement.

**Figure 1:**
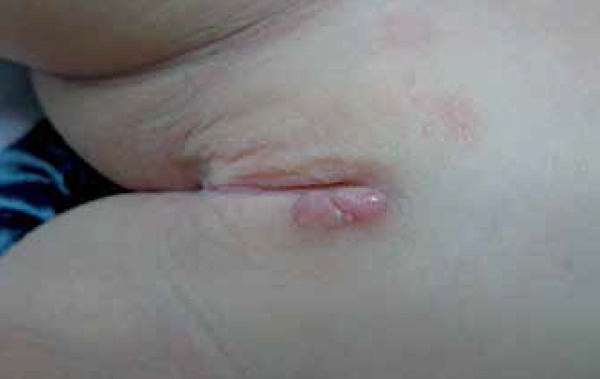
Tumor with smooth surface, fibroelastic consistency, discreetly erythematous, well delimited, measuring about 3cm on the left labium majus

At the dermatological examination, she presented a erythematous nodule with smooth surface, fibroelastic consistency, measuring about 3cm, on the left labium majus (Figure 1).

**Figure 2:**
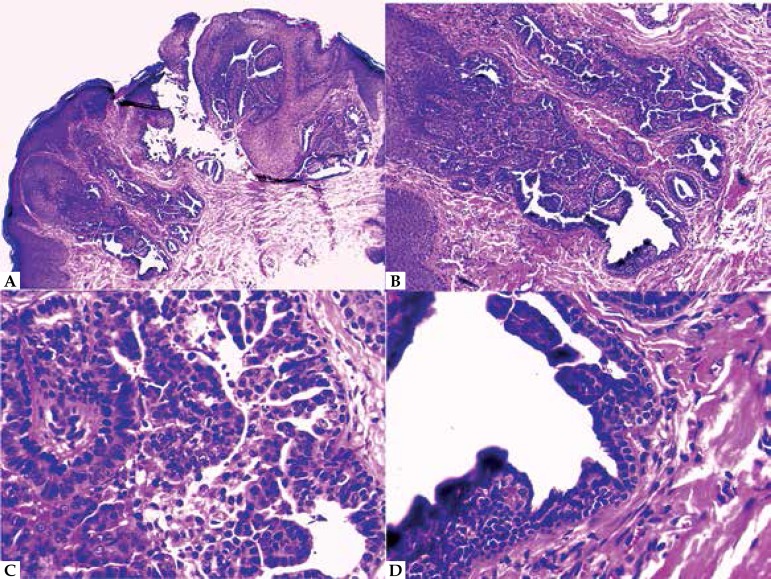
A and B: Anatomopathological examination of lesion biopsy stained with hematoxilyn and eosin showed papillomatous epidermis presenting multiple papillary cystic invaginations which extended to the dermis (HEx10). C: Detail of glandular epithelium constituted by an external layer of cuboid cells, with round nuclei and scarce cytoplasm, and an internal layer of cylindrical cells with decapitation secretion (HEx40) D: Detail of decapitation secretion and a plasmocyte-rich inflammatory infiltrate (HE x40)

The anatomopathological examination of biopsy of the lesion showed papillomatous epidermis associated with invaginations and papillary projections. Glandular epithelium presented as an external layer of cuboid cells, with round nuclei and scarce cytoplasm, and an internal layer of cylindrical cells with decapitation secretion. The dermis revealed a plasmocyte-rich inflammatory infiltrate. These findings are compatible with SCAP (Figure 2). The patient was referred to pediatric surgery for surgical excision.

## DISCUSSION

SCAP is a hamartomatous adnexal tumor which arises from pluripotent cells. Derived from apocrine and eccrine glands, its histogenesis is still controversial.^1,2,3^ It is accepted that differentiation is predominantly apocrine^1^. It is a childhood or adolescence neoplasm, observed since birth in 50% of cases, as in the present case.

Its clinical presentation is a papule, plaque or a single or grouped nodule, with no hairs, asymptomatic, but that may become exsudative and with a linear aspect arrangement.^1-4^It has a tendency to increase in size during adolescence, becoming more verrucous and papillomatous.^1,3^ Its most frequent sites are the scalp, neck and face (75%), with already reported uncommon sites: eyelids, arms, buttocks, auricular pavilion, scrotum, vulva, back and abdomen which are very rare locations, such as the one presented in this case.^2-5^

Association with other benign neoplasms of adnexal origin may occur, such as apocrine adenoma, hidradenomapapilliferum, trichoblastoma, eccrine poroma, sebaceous nevus of Jadassohn and others.^1,2,4^Histopathologically, its presentation is a dermal endophytic tumor with irregular papillary projections of scaly epithelium, forming ductile structures which connect with the surface, aligned by glandular epithelium constituted by an external layer of cuboid cells, with round nuclei and scarce cytoplasm, and an internal layer of cylindrical cells with decapitation secretion and plasmocyte-rich inflammatory infiltrate.^1,2^

Tumor cells show a reaction of positive staining with carcinoembryonic antigen. It is rarely associated with malignant progression, but basal-cell carcinoma development was described in 10% of the cases, mainly when associated with sebaceous nevus of Jadassohn. Squamous cell carcinoma and syringocystadenocarcinoma papilliferum were also reported as a progression of SCAP, but are extremely rare. Healing treatment is surgical excision, but there are cases in which removal was effective with CO_2_ laser in locations unfavorable for surgery.^3,4^

The patient in the present case was referred to pediatric surgery, because excision is recommended to prevent infections, hemorrhages, exacerbated growth and malignant degeneration.^1,4,6^ SCAP is a rare benign adnexal neoplasm which present few reports of uncommon location described in the literature. This illustrates an additional case of atypical location of this rare disease and contributes to the differential diagnosis of vulvar lesions.
